# Draft Genome Sequence of Streptomyces sp. Strain RFCAC02, Isolated from the Gut Microflora of the Pacific Chub Mackerel Scomber japonicus peruanus

**DOI:** 10.1128/MRA.00355-19

**Published:** 2019-06-06

**Authors:** Wilbert Serrano, Raul M. Olaechea, Joachim Wink, Michael W. Friedrich

**Affiliations:** a Laboratorio de Microbiología Molecular y Genómica Bacteriana, Dirección General de Investigación Desarrollo e Innovación, Universidad Científica del Sur, Lima, Peru; b Microbial Ecophysiology Group, Faculty of Biology/Chemistry, University of Bremen, Bremen, Germany; c MARUM, Center for Marine Environmental Sciences, University of Bremen, Bremen, Germany; d Department of Microbial Natural Products, Helmholtz Centre for Infection Research, Braunschweig, Germany; Georgia Institute of Technology

## Abstract

A new strain of Streptomyces sp., strain RFCAC02, was isolated from the gut of the Pacific chub mackerel Scomber japonicus peruanus. This strain produces a variety of secondary metabolites. Further bioinformatic analysis revealed the presence of biosynthetic gene clusters putatively coding for compounds related to the polycyclic tetramate macrolactams (PTMs).

## Abstract (precis)

A new strain of Streptomyces sp., strain RFCAC02, was isolated from the gut of the Pacific chub mackerel Scomber japonicus peruanus. This strain produces a variety of secondary metabolites.

## ANNOUNCEMENT

The uncontrolled use of antibiotics has created conditions for the emergence of multidrug-resistant pathogenic microorganisms, which is challenging health systems worldwide. Particularly relevant are clinical strains of the Enterococcus faecium, Staphylococcus aureus, Klebsiella pneumoniae, Acinetobacter baumannii, Pseudomonas aeruginosa, and Enterobacter species (ESKAPE) group (1). Aside from bacterial infections, another risk to human health comes from invasive fungal diseases (IFDs), opportunistic infections that overwhelm critically ill or immunosuppressed patients (2). Sources of antifungal metabolites, e.g., terpenoid, phenolic compounds, and phytoalexins, are mainly obtained from plants (3), although some others, such as echinocandins, originate from saprophytic fungal species (4). Additionally, some members of the genera Bacillus, *Pseudomonas*, Proteus, *Staphylococcus*, and Streptomyces are well-known producers of antifungal secondary metabolites (5, 6). Together with bacterial infections, fungus-caused diseases have increased dramatically in the last decades (7). Therefore, the search for novel antifungal metabolites derived from diverse sources is an urgent need.

Strain RFCAC02 was isolated from the homogenized stomach contents of the marine fish Scomber japonicus peruanus. Briefly, homogenates were incubated at 60°C for 25 minutes. Aliquots of the homogenate were serially diluted in sterile sodium chloride solution (0.9%), inoculated on oatmeal ISP3 agar plates (8), and incubated for 7 days at 30°C. The resulting pure culture was identified as a member of the genus *Streptomyces* by 16S rRNA gene sequence analysis. Strain RFCAC02 showed 97.5% similarity to Streptomyces avicenniae strain MCCA 1A01535. Genomic DNA was extracted using a phenol-chloroform method, according to Marmur (9); for this purpose, strain RFCAC02 was cultivated in liquid DSMZ 1159 medium containing glucose-yeast extract-malt extract (GYM) supplemented with 10% NaCl for 7 days at 30°C and 200 rpm. Sequencing was accomplished using the single-molecule real-time (SMRT) technology (10) on a PacBio RS II system (Pacific Biosciences, USA) by Macrogen, Inc., South Korea. The number of polymerase reads obtained by the system was 43,556, with an average length of 13,505 bp. *De novo* assembly was performed using the Hierarchical Genome Assembly Process (HGAP) (11) (expected genome length set to 6,500,000 bp) within SMRT Link (smrtlink-release_6.0.0.47841; Pacific Biosciences), using 81,365 subreads with an average length of 7,011 bp. A final genome of 6.15 Mbp in two scaffolds of 6,130,205 and 23,083 bp, with a G+C content of 73.5% and 76× sequence coverage was obtained. The assembled sequence was annotated using the Rapid Annotations using Subsystems Technology (RAST) v2.0 server (http://rast.nmpdr.org) (12), which predicted 5,850 coding sequences, of which 2,549 encoded hypothetical proteins and 64 encoded noncoding RNAs.

Species circumscription of strain RFCAC02 was achieved using the online server JSpeciesWS (13). Thus, pairwise genome comparison confirmed a close relationship between strain RFCAC02 and *S. avicenniae* MCCC 1A01535, showing an average nucleotide identity (ANI) of 80.58%, a value below the accepted cutoff threshold for species delimitation of 95% (14). Therefore, strain RFCAC02 might represent a new species within the genus *Streptomyces*. An antiSMASH v4.0 search (15) revealed the presence of 20 putative biosynthetic gene clusters, including clusters coding for the production of polycyclic tetramate macrolactams (PTMs). PTMs are a family of biomedically promising natural products and merit further studies.

### Data availability.

This whole-genome shotgun project has been deposited at DDBJ/ENA/GenBank under accession number SAUH00000000. The version described in this paper is version SAUH01000000. The raw sequencing data are available in the Sequence Read Archive (SRA) database under accession number SRX5604831.
